# Editorial: Cancer Diagnostic and Therapeutic Target Identification and Verification Based on the Regulatory Functions of MicroRNAs

**DOI:** 10.3389/fgene.2017.00178

**Published:** 2017-11-15

**Authors:** Lawrence W. C. Chan, S. C. C. Wong

**Affiliations:** Department of Health Technology and Informatics, Hong Kong Polytechnic University, Kowloon, Hong Kong

**Keywords:** microRNA, cancer, diagnostics, therapeutics, bioinformatics, *in-vitro* bioassay, proteomics

As short (21–25 nucleotides), endogenous and non-coding RNA fragments, MicroRNAs (miRNAs) regulate target gene expression by mediating mRNA degradation or inhibiting protein translation through their binding to the complementary sequence motifs in 3′-untranslated region of the gene transcripts, which was first identified in *Caenorhabditis elegans* (Lee et al., 1993). miRNAs have been implicated in many signal circuits in various diseases, including lung cancer and leukemia. According to 2017 fact sheet of World Health Organization, lung cancer is the most common cause of cancer death (WHO, 2017). Non-small cell lung cancer (NSCLC) accounts for 85% of lung cancer cases and the mortality is high due to the late diagnosis and poor prognosis. As early detection of NSCLC becomes very crucial, many studies have been performed to investigate diagnostic biomarkers for NSCLC, showing significant expressional differences between cancer tissues or cell lines compared to healthy controls. In this research topic, a review presented the current knowledge about the mechanism of miRNAs release into bloodstream, the cell-free miRNAs as the circulating NSCLC biomarkers, and their limitations (Hou et al.). Circulating tumor cell (CTC) is useful “liquid biopsy” for NSCLC diagnosis as it facilitates structural evaluation and molecular characterization of cancer phenotype (Calabuig-Fariñas et al., 2016). CTC-derived or -associated miRNAs would provide important information about the tumor. Several issues remain unclear in the field of enrichment and detection of CTC and miRNAs.

Besides the roles of oncogenes or tumor suppressor genes in human cancer, miRNAs may alter the acquired drug resistance and tumor progression under targeted therapy. Bioinformatics, cell line experiments, and proteomics analysis are very valuable for advancing the identification and verification of cancer diagnostic and therapeutic targets. A bioinformatics research article identified the association between the expression levels of the resistance-related genes and that of their predicted targeting microRNAs in samples collected from patients (Wang et al.). The direct EGFR downstream molecules in the EGFR signaling pathway shared by other TKRs were selected for further analysis. From the microarray data, the significant and principal miRNAs regulating the mRNAs of these molecules were found using the mRNA:miRNA multiple linear regression analysis. The regression analysis results showed that miR-34a was significantly negatively associated with PLCG1, miR-30a-5p was significantly negatively associated with PIK3R2, miR-27a was significantly negatively associated with GRB2, miR-302b was significantly positively associated with JAK1, and miR-520e was significantly negatively associated with JAK1, as well as miR-155 was significantly positively associated with JAK2. Due to the important role of phosphoinositide-3-kinase (PI3K) in cancer cell growth, proliferation, differentiation, motility, survival, and intracellular trafficking, miR-30a-5p represents a good candidate for further *in-vitro* analysis.

Genes downstream of the signaling pathway shared between EGFR and other TKRs and the associated miRNAs may play an important role in EGFR TKI response in NSCLC (Camp et al., 2005). A study examined whether the expression manipulation of these genes and miRNAs reduces the resistance to EGFR TKI in NSCLC cell lines (Meng et al.). The *in-vitro* study considered three potential treatment options, EGFR inhibitor (Gefitinib), IGF-1R inhibitor (AEW541), and miRNA mimics, and two gefitinib-resistant NSCLC cell lines, NCI-H1975 (acquired a secondary mutation T790M of EGFR), and NCI-H460. Gefitinib and AEW541 were simultaneously applied to examine the inhibition effect on the overlapping signaling pathways. It was found that the combination of Gefitinib and AEW541 inhibitors significantly reduced p-EGFR, p-IGF-1R, and p-AKT expression levels compared to Gefitinib, AEW541, and control groups in both H460 and H1975 cells. As AKT is the downstream molecule in the signaling pathways, and the expression level of its phosphorylated form was the lowest in the dual inhibitors group compared to the other three groups, the combination of EGFR and IGF-1R inhibitors treatment could block the PI3K/AKT signaling pathway. To show the function of miRNAs as multi-target regulators, the NSCLC cells were transfected with MiR-30a-5p mimics and control using Lipofectamine reagent and then treated with Gefitinib and vehicle. The resulted samples were assessed using the western blotting, cell apoptosis, wound healing, and invasion assays. The treatment with miR-30a-5p mimics significantly reduced the expression level of PIK3R2 and p-AKT compared to the control group. The significant effect of miR-30a-5p mimics on the apoptosis, invasion, and migration was also demonstrated in H460 and H1975 cells.

As an E3 ubiquitin ligase, the seven in absentia homolog 2 (SIAH2) mediates ubiquitination and subsequent proteasomal degradation of its target proteins. The p38 mitogen-activated protein kinase (MAPK) represents a downstream protein of EGFR pathway other than AKT and PI3K. It is interesting that major phosphorylation site of SIAH2 is conserved for p38 MAPK phosphorylation (Khurana et al., 2006). In a study of 152 surgically resected primary NSCLC samples, SIAH2 expression is correlated with the clinically-defined tumor grade (Moreno et al., 2015). PI3K affects the TKI sensitivity. However, it remains unclear how PI3K and SIAH2 are related and whether they can be regarded as treatment co-targets and theranostic dual markers for overcoming TKI resistance. It was found that a group of microRNAs in miR-30 family concurrently regulate PI3K and SIAH2 (Chan et al.). Mass spectrometry data analysis showed that SIAH2 expression is upregulated in NSCLC. Bioinformatics analyses explored its indirect interaction among SIAHS, PI3K, and predicted their targeting microRNAs in common. The potential role of miR-30 family was explored in the modulation of PI3K-SIAH2 interaction in NSCLC.

Target genes regulated by the same miRNA tend to be co-expressed. A study focused on the miR-17-92 cluster whose miRNAs have an oncogenic activity in chronic myelogenous leukemia (CML) patients compared with normal individuals (Wang et al.). The difference between the normal and the CML groups in the co-expression patterns of those genes directly regulated by miR-17-92 cluster was explored. Functional annotation for co-expressed gene pairs indicated the stronger involvement of metabolism processes in normal than CML. The co-expression pattern observed in the normal group can be regarded as the inter-gene linkages under healthy pathological balance. Conversely, the weakly co-expressed profiles of miR-17-92 targeted genes can be a marker of CML.

## Author contributions

LWCC is responsible for organizing the materials and writing this editorial. SCCW is responsible for proof-reading this editorial.

### Conflict of interest statement

The authors declare that the research was conducted in the absence of any commercial or financial relationships that could be construed as a potential conflict of interest.
